# Bilateral Transankle Intervention as a Critical Determinant of Successful Revascularisation in Complex Peripheral Arterial Disease

**DOI:** 10.1016/j.ejvsvf.2024.07.038

**Published:** 2024-07-26

**Authors:** Tetsuya Nomura, Naotoshi Wada, Kenshi Ono, Keisuke Shoji

**Affiliations:** Department of Cardiovascular Medicine, Kyoto Chubu Medical Centre, Kyoto, Japan

**Keywords:** Transankle intervention, Endovascular treatment, Critical limb threatening ischaemia, Intravascular ultrasound, Parallel wiring

## Abstract

**Introduction:**

Establishing optimal vascular access sites is important for the procedural success of endovascular treatment (EVT) and the patient's comfort afterwards. Among the variety of vascular access sites, the transankle intervention (TAI) has been used more recently; however, there have been no reports of complex lower extremity arterial disease lesions treated with the TAI manoeuvre.

**Report:**

An 82 year old man with chronic limb threatening ischaemia in both lower extremities underwent EVT for bilateral long segment occlusion from the iliac arteries to the superficial femoral artery (SFA). The right posterior tibial artery was punctured under extravascular ultrasound guidance and a Parent Select 5082 guide sheath was inserted. The guidewire was manipulated under intravascular ultrasound (IVUS) guidance. When the first guidewire entered the subintimal space, the second guidewire was manipulated to advance through the intraplaque route, while monitoring it using IVUS. The intraluminal space of the right common iliac artery was reached by repeating these procedures. A self expandable stent was deployed in the external iliac artery and drug coated balloons were inflated from the common femoral artery to the SFA; good vascular patency and favourable blood flow were confirmed. Subsequently, a similar TAI procedure was performed from the left dorsalis pedis artery, and successful revascularisation was achieved from the left common iliac artery to the SFA. After revascularisation, the persistent pain disappeared in the right lower limb and the wound healed favourably in the left lower limb.

**Conclusion:**

In this case of complex chronic limb threatening ischaemia, the TAI strategy worked favourably for successful revascularisation. Transankle intervention can provide various advantages for successful EVT.

## Highlights

• Optimal vascular access sites are important for successful endovascular treatment.
• The concept of transankle intervention (TAI) has been developed more recently.
• TAI can provide various advantages for both operators and patients.
• Arterial occlusion of the puncture site must be carefully noted.
• This case may pave the way for TAI in complex endovascular treatment.

## Introduction

Techniques and devices for endovascular treatment (EVT) of lower extremity arterial disease (LEAD) are regularly being developed, resulting in higher revascularisation success rates. Endovascular treatment begins with the establishment of vascular access sites. Optimal vascular access sites are significantly involved in the procedural success of EVT and the patient's comfort after EVT. Pioneers have developed the transankle intervention (TAI), and experience and knowledge of this new EVT strategy are being accumulated.

## Case Report

**Figure 1:**
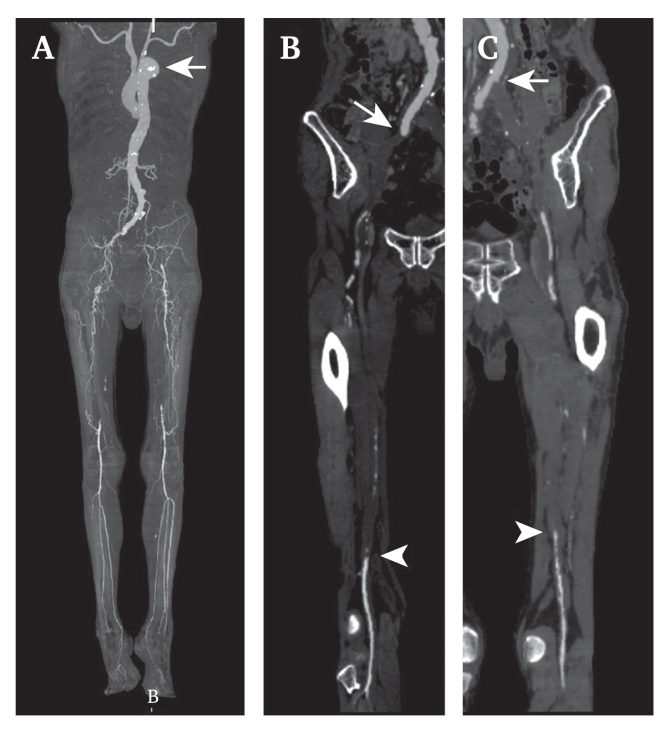
Computed tomography angiography and curved planar reconstruction images. (A) Computed tomography angiography showing long segment arterial occlusions on both sides and thoracic aneurysm formation at the aortic arch (arrow). (B) Curved planar reconstruction image of the artery in the right lower extremity, with the arrow pointing to the start and the arrowhead pointing to the end point of the occlusion. (C) Curved planar reconstruction image of the artery on the left lower extremity, with the arrow pointing to the start and the arrowhead pointing to the end point of the occlusion.

An 82 year old man without a medical history was admitted for treatment of chronic limb threatening ischaemia in his lower extremities (Rutherford 4 on the right and Rutherford 5 on the left sides). The Wound Ischaemia and foot Infection classification was stage 2 with W-0, I-3, and fI-0 on the right side and stage 3 with W-1, I-3, and fI-0 on the left side. The ankle brachial pressure index was unmeasurable on both sides. Computed tomography angiography (CTA) indicated occlusions from the external iliac artery (EIA) to the distal superficial femoral artery (SFA) on the right side and from the ostium of the common iliac artery (CIA) to the distal SFA on the left. The deep femoral arteries looked to be supplying favourable collateral arterial flow on both sides. The infrapopliteal arteries appeared patent, except for the right anterior tibial artery on CTA (Fig. 1A–C). A thoracic arterial aneurysm was seen in the aortic arch (Fig. 1A, arrow); it was classified as stage III based on the Global Limb Anatomic Staging System, with femoropopliteal grade 4 and infrapopliteal grade 0 on both sides.^1^ Because an optimal great saphenous vein for bypass grafting was unavailable for this patient and he strongly wanted less invasive treatment, EVT was implemented for limb salvage and it was decided to adopt the TAI strategy due to the complex pathology.

**Figure 2:**
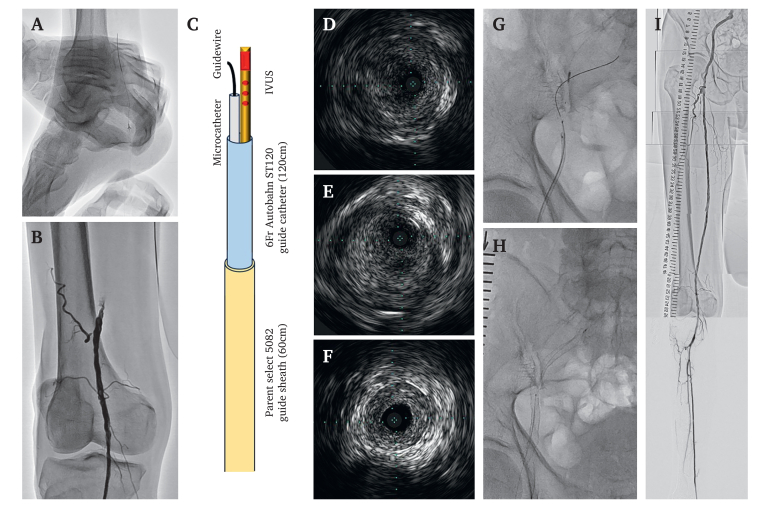
Endovascular treatment procedure. (A) A guidewire was inserted after puncturing the posterior tibial artery with extravascular ultrasound (EVUS) guidance. (B) The initial angiogram showed the closed stump of the superficial femoral artery occlusion. (C) An illustration of the intravascular ultrasound (IVUS) guided parallel wiring system representing the IVUS short axis images of the guidewire passing route. (D) External iliac artery. (E) Common femoral artery. (F) Superficial femoral artery. (G) Second guidewire advance through the intraplaque route with IVUS monitoring. (H) Deployment of a SMART self expandable stent in the right external iliac artery. (I) Final angiogram confirming vascular patency and favourable blood flow in the right lower extremity.

Two days before catheterisation, 100 mg of aspirin and 75 mg of clopidogrel were prescribed daily. The right posterior tibial artery was punctured under extravascular ultrasound (EVUS) guidance with local anaesthesia. A guidewire was then advanced into the posterior tibial artery (Fig. 2A) and a Parent Select 5082 (60 cm) guide sheath (MEDIKIT, Tokyo, Japan) was inserted. Immediately after guide sheath insertion, an intra-arterial bolus of 5 000 units of unfractionated heparin was administered. The activated clotting time was controlled to ≥250 seconds during the procedure. Initial angiography via the retrograde guide sheath indicated the closed stump of the SFA occlusion (Fig. 2B). A 6 F Autobahn ST120 guide catheter (NIPRO, Osaka, Japan) (2.10 mm outer diameter) was prepared in the Parent Select 5082 guide sheath (2.12 mm inner diameter) and a 0.014 inch Gladius MG guidewire (ASAHI INTECC, Aichi, Japan) was advanced into the occlusion under Eagle Eye Platinum ST (PHILIPS, Amsterdam, Netherlands) IVUS guidance. When the first guidewire advanced into the subintimal space, a 0.014 inch Halberd guidewire (ASAHI INTECC, Aichi, Japan) was delivered with a Prominent AS microcatheter (Tokai Medical Products, Aichi, Japan) while remaining in the IVUS catheter; Fig. 2C illustrates the IVUS guided parallel wiring system. Subsequently, the Halberd guidewire was manipulated to advance through the intraplaque route by IVUS monitoring (Fig. 2D–G). By repeating these sequential procedures, the intraluminal space of the right CIA was reached. Intravascular ultrasound showed that most of the occluded lesions were filled with organised thrombi, which suggested that the aetiology was due to a chronic condition following acute limb ischaemia (Fig. 2D–F).^2^ After lesion preparation with optimal balloon dilation (EIA: 6.0/60 mm, SFA: 5.0/300 mm), a 8.0/100 mm SMART self expandable stent (Cordis, FL, USA) was deployed in the EIA (Fig. 2H) and the operation was finished with two Ranger drug coated balloon (Boston Scientific, MA, USA) (5.0/200 mm, +6.0/200 mm) dilations from the common femoral artery to the SFA. Good vascular patency and favourable blood flow were confirmed with IVUS and angiography (Fig. 2I). The procedure time was 149 minutes, and the absorbed radiation and effective radiation doses were 0.1104 Gy and 9 μSv, respectively.

**Figure 3:**
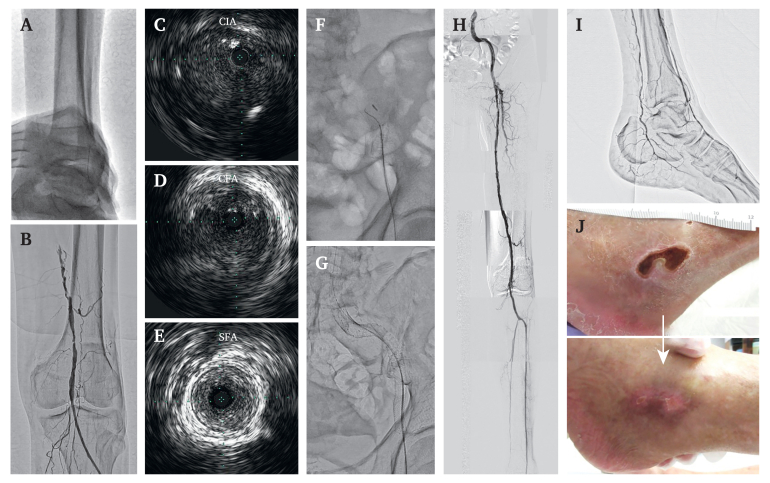
Transankle intervention for long segment occlusive disease on the left side. (A) A guidewire was inserted after puncturing the dorsalis pedis artery with extravascular ultrasound guidance. (B) Initial angiography showing the exit of the superficial femoral artery occlusion, representing intravascular ultrasound short axis images of the guidewire passing route. (C) Common iliac artery. (D) Common femoral artery. (E) Superficial femoral artery. (F) Repeated parallel wiring to trace the intraplaque route. (G) Deployment of three SMART stents from the common iliac artery ostium to the external iliac artery. (H) Final angiogram confirming vascular patency and favourable blood flow in the left lower extremity. (I) Confirmation of the intact left dorsalis pedis artery 19 days after the second intervention. (J) Wound healing in the left lower limb 35 days after the second intervention.

Nine days after EVT on the right side, TAI was performed for long segment occlusive disease on the left side. The left dorsalis pedis artery was punctured under EVUS guidance and a Parent Select 5082 guide sheath was inserted (Fig. 3A and B). The procedures were similar to those performed on the right side. The guidewire was passed retrogradely through the long segment occlusion from the distal SFA to the ostial CIA, all in the intraplaque route (Fig. 3C–F). After lesion preparation with optimal balloon dilation (EIA: 5.0/40 mm, SFA: 6.0/300 mm), three SMART self expandable stents (8.0/60 mm, +10.0/40 mm, +10.0/60 mm) were deployed from the ostial CIA to the EIA (Fig. 3G). Three IN.PACT Admiral drug coated balloons (Medtronic, MN, USA) (6.0/200 mm, +6.0/150 mm, +6.0/80 mm) were inflated from the common femoral artery to the SFA. Good vascular patency and favourable blood flow were confirmed with IVUS and angiography (Fig. 3H). The procedure time was 210 minutes, and the absorbed radiation and effective radiation doses were 0.1335 Gy and 10 μSv, respectively. PreludeSYNC (MERIT MEDICAL, UT, USA) haemostat band fixing was used for two hours to ensure optimal TAI haemostasis on both sides.

The patient did not require bed rest immediately after TAI. After revascularisation, the ankle brachial pressure index had increased to 0.98 and 1.11 on the right and left sides, respectively. Administration of dual antiplatelet therapy was continued after the intervention and scheduled for three months. Nineteen days after the second EVT session, the lower extremity angiography was checked when conducting coronary intervention from the upper extremity; it confirmed that the punctured dorsalis pedis artery was patent (Fig. 3I). The persisting pain in the right lower limb disappeared, and the wound healed favourably in the left lower limb 35 days after the second EVT session (Fig. 3J). The patient was discharged to an elderly care home about one month later and was walking independently.

## Discussion

Endovascular treatment was developed using the transfemoral approach, which has been the gold standard since the beginning of catheterisation. However, this approach has shortcomings, such as necessitating post-procedural bed rest or possible bleeding complications, which sometimes become fatal. Thus, pioneers have developed the transradial approach to peripheral catheterisation, which has gradually become more popular.^3^^,^^4^ However, transradial EVT is not preferable for treating complex LEAD lesions because of the difficulty in manipulating the system and the limited size variation of devices. A safe procedure using this system depends on condition of the aorta, such as an aortic aneurysm or a shaggy aorta. On the other hand, multiple distal puncture sites have demonstrated safety and efficacy in clinical settings.^5^^,^^6^ However, the retrograde EVT system based on this kind of distal puncture usually only consists of a microcatheter, which also means that an antegrade approaching system is essential for this strategy.

There are several reports on tibiopedal arterial minimally invasive retrograde revascularisation (TAMI).^7^ In this technique, a 4 F regular sheath is used for the vascular access route and only balloon angioplasty is conducted in most cases. Unlike this procedure, the concept of TAI has emerged because of the evolution of slender guide sheath devices. The Parent Select 5082 guide sheath can easily be inserted into both tibial arteries; it functions well for delivery of various EVT devices, including stents, and of EVT technique performance, suggesting that EVT procedures can be completed using only a retrograde approach. In contrast to transradial EVT or the TAMI technique, which is not preferable for treating complex lesions, TAI is relatively useful for treating complex EVT. This is because most LEAD lesions, such as the aorto-iliac or femoropopliteal areas, can easily be accessed, and EVT devices are easy to be intentionally manipulated due to their straight design. Distal embolism that happens during the procedure is easy to deal with because of the distal arrangement of the guide sheath. Furthermore, it is possible that TAI can reduce the operator's self-radiation exposure compared with transradial or transfemoral EVT. Haemostasis at the puncture point is easily achieved by setting the dedicated haemostat band. These TAI characteristics are considered operator friendly.

However, there are disadvantages to this method. Initial angiography from upstream is difficult; therefore, clinicians should obtain overall images, such as CTA, in advance. Guide sheath insertion poses a risk of injury to the punctured artery; therefore, this manoeuvre should be avoided for the only remaining infrapopliteal artery. On the other hand, according to a previous report that examined IVUS guided interventions for below the knee disease, the mean balloon size for IVUS guided procedures was relatively large (2.45 ± 0.4 mm) in diameter, which suggests that infrapopliteal arteries are much larger than expected from the angiographic assessment.^8^ The Parent Select 5082 guide sheath is 2.40 mm in outer diameter, which is considered not much larger than the average size of the infrapopliteal arteries. Haemostasis management to maintain arterial flow distally from the puncture point is more important. Final angiography is performed immediately before guide sheath removal; therefore, distal runoff of the punctured artery cannot be confirmed at that time. In this regard, EVUS of the punctured artery is usually checked after finishing haemostasis. Doppler flow distal to the puncture point was observed in this case; the punctured dorsalis pedis artery was angiographically confirmed to be intact on the left side after this.

The history of TAI remains too short to establish the effectiveness and safety of this novel EVT concept. However, TAI is considered to have various advantages for successful a EVT procedure. To make the TAI procedure more sophisticated, experience and knowledge of this technique must be collected. Again, it is very important to select cases in which the TAI strategies can be safely managed. In this case of chronic limb threatening ischaemia with bilateral long segment LEAD, the TAI strategy selection was a critical determinant of successful revascularisation. Complex LEAD lesions have not previously been treated with the TAI manoeuvre, and this novel case may pave the way for TAI for complex EVT.

## Conflict of interest

The authors declare that they have no competing interests.

## Funding

Not applicable.
